# Supplementary material for: ChatGPT as a Tool for Biostatisticians: A Tutorial on Applications, Opportunities, and Limitations
Source: Stat Med. 2025 Oct 23;44(23-24):e70263. doi: 10.1002/sim.70263 (PMC12548020; doi:10.1002/sim.70263)
Supplement: Supplementary file 1 — Data S1. Supporting Information. [file SIM-44-0-s001.zip › Supplementary Materials Additional.zip!/3.1_Systematic_Reviews_and_Meta_aspects/3.1_Forest Plot - 4o - Chat 5.pdf]

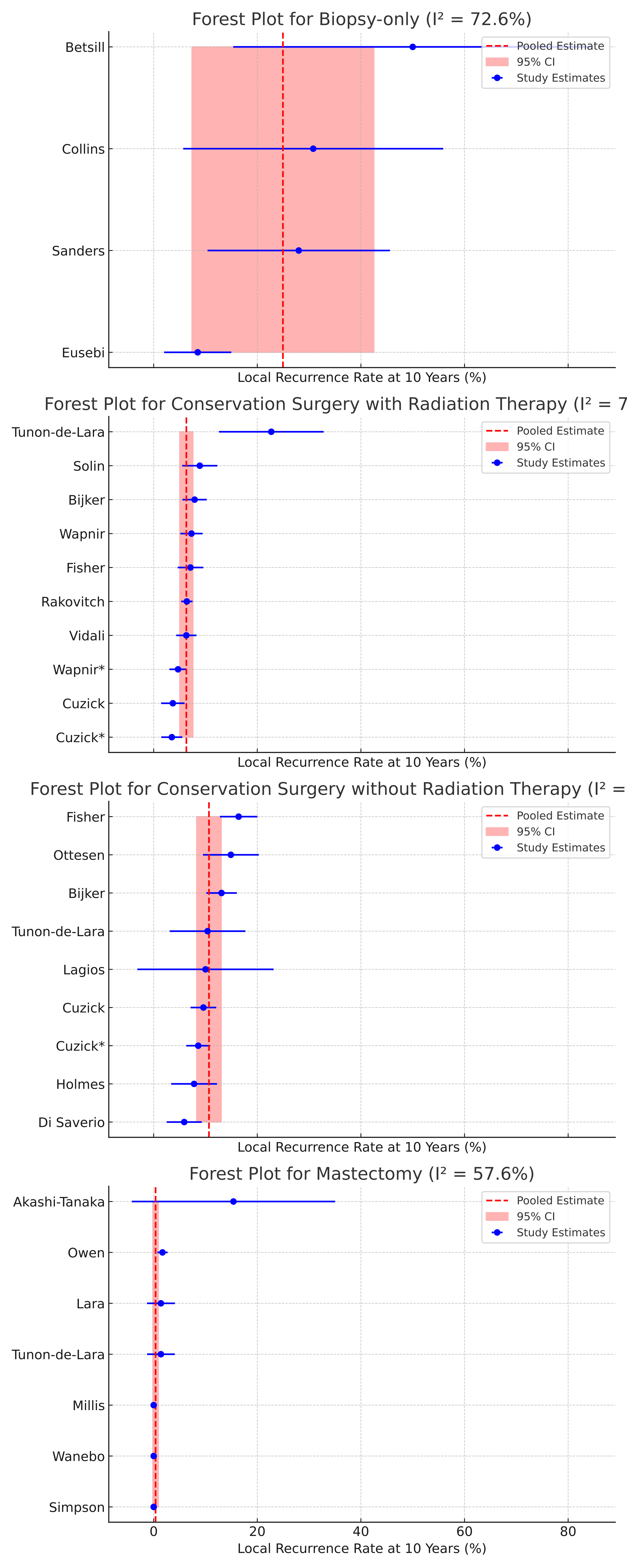
Forest Plot for Biopsy-only (I² = 72.6%)
Betsill
Collins
Sanders
Eusebi
Pooled Estimate
95% CI
Study Estimates
Local Recurrence Rate at 10 Years (%)
Forest Plot for Conservation Surgery with Radiation Therapy (I² = 7
Tunon-de-Lara
Solin
Bijker
Wapnir
Fisher
Rakovitch
Vidali
Wapnir*
Cuzick
Cuzick*
Pooled Estimate
95% CI
Study Estimates
Local Recurrence Rate at 10 Years (%)
Forest Plot for Conservation Surgery without Radiation Therapy (I² =
Fisher
Ottesen
Bijker
Tunon-de-Lara
Lagios
Cuzick
Cuzick*
Holmes
Di Saverio
Pooled Estimate
95% CI
Study Estimates
Local Recurrence Rate at 10 Years (%)
Forest Plot for Mastectomy (I² = 57.6%)
Akashi-Tanaka
Owen
Lara
Tunon-de-Lara
Millis
Wanebo
Simpson
Pooled Estimate
95% CI
Study Estimates
0
20
40
60
80
Local Recurrence Rate at 10 Years (%)
